# Dysfunctional Cortical Gradient Topography in Treatment-Resistant Major Depressive Disorder

**DOI:** 10.1016/j.bpsc.2022.10.009

**Published:** 2022-10-31

**Authors:** Lorenzo Pasquini, Susanna L. Fryer, Stuart J. Eisendrath, Zindel V. Segal, Alex J. Lee, Jesse A. Brown, Manish Saggar, Daniel H. Mathalon

**Affiliations:** Department of Neurology, University of California, San Francisco, San Francisco, California; Department of Psychiatry and Behavioral Sciences, University of California, San Francisco, San Francisco, California; San Francisco Veteran Affairs Health Care System, San Francisco, California; Department of Psychiatry and Behavioral Sciences, University of California, San Francisco, San Francisco, California; Department of Psychiatry and Behavioral Sciences, University of California, San Francisco, San Francisco, California; Department of Neurology, University of California, San Francisco, San Francisco, California; Department of Neurology, University of California, San Francisco, San Francisco, California; Department of Psychiatry and Behavioral Sciences, Stanford University, Stanford, California; Department of Psychiatry and Behavioral Sciences, University of California, San Francisco, San Francisco, California

## Abstract

**BACKGROUND:**

Treatment-resistant depression (TRD) refers to patients with major depressive disorder who do not remit after 2 or more antidepressant trials. TRD is common and highly debilitating, but its neurobiological basis remains poorly understood. Recent neuroimaging studies have revealed cortical connectivity gradients that dissociate primary sensorimotor areas from higher-order associative cortices. This fundamental topography determines cortical information flow and is affected by psychiatric disorders. We examined how TRD impacts gradient-based hierarchical cortical organization.

**METHODS:**

In this secondary study, we analyzed resting-state functional magnetic resonance imaging data from a mindfulness-based intervention enrolling 56 patients with TRD and 28 healthy control subjects. Using gradient extraction tools, baseline measures of cortical gradient dispersion within and between functional brain networks were derived, compared across groups, and associated with graph theoretical measures of network topology. In patients, correlation analyses were used to associate measures of cortical gradient dispersion with clinical measures of anxiety, depression, and mindfulness at baseline and following the intervention.

**RESULTS:**

Cortical gradient dispersion was reduced within major intrinsic brain networks in patients with TRD. Reduced cortical gradient dispersion correlated with increased network degree assessed through graph theory–based measures of network topology. Lower dispersion among default mode, control, and limbic network nodes related to baseline levels of trait anxiety, depression, and mindfulness. Patients’ baseline limbic network dispersion predicted trait anxiety scores 24 weeks after the intervention.

**CONCLUSIONS:**

Our findings provide preliminary support for widespread alterations in cortical gradient architecture in TRD, implicating a significant role for transmodal and limbic networks in mediating depression, anxiety, and lower mindfulness in patients with TRD.

Major depressive disorder (MDD) is a common, debilitating disorder and is among the leading causes of disability worldwide (1). Although several treatment options are available for depression, a significant number of patients do not improve despite adequate antidepressant trials (2). Patients who, after repeated treatments, do not reach acceptable levels of functioning and well-being eventually present with treatment-resistant depression (TRD), a condition associated with a significant social and economic burden (2,3). TRD is often defined as the failure to remit after at least 2 antidepressant trials of adequate dose and duration (2,3). A consensus characterization of TRD, however, has yet to be achieved, partly due to a poor understanding of its neurobiological basis and a lack of reliable diagnostic biomarkers (4,5).

Resting-state functional magnetic resonance imaging (rs-fMRI) is a neuroimaging modality commonly used to measure functional connectivity of brain networks in terms of correlated spontaneous activity among distant brain regions (6,7). This method has proven useful in revealing altered functional connectivity within and between large-scale brain networks in depression (5,8–12). Crucially, brain network dysfunctions in MDD primarily affect limbic and higher-order associative systems including the default mode network (DMN) (10,13,14), control network (CoN) (5,8–12), and limbic network (LiN) (5,8–12), with imbalances in these systems being linked to emotional dysregulation and maladaptive self-referential processes, such as rumination (9,15,16).

Fundamental principles in behavioral neurology and recent neuroimaging studies provide convergent support for a hierarchical cortical organization that separates primary sensorimotor systems from transmodal associative areas (17–19). Cortical microstructure, connectivity, and gene expression findings point to dominant sensorimotor-to-transmodal gradients organizing the propagation of sensory inputs from primary areas into transmodal regions along multiple cortical relays (17,18,20). This large-scale brain system organization anchors the DMN at one end of the hierarchy with respect to primary sensorimotor areas, capturing a functional topography that enables the transition from perception to more abstract cognitive functions (9,15,16). Several neuropsychiatric disorders, including MDD (21), cognitive vulnerability to depression (22), and autism (20), profoundly impact connectivity-based cortical gradient organization. MDD also disrupts global topography by producing focal alterations of cortical gradients among primary sensory and transmodal regions involved in high-order cognitive processing (21).

Accordingly, we hypothesized that TRD would impact hierarchical brain network organization and that functional deficits affecting the DMN, CoN, and LiN would predict baseline and future symptoms of depression following group treatment with either mindfulness-based cognitive therapy (MBCT) or a health enhancement program (HEP). We retrospectively applied recently developed gradient decomposition techniques (23) to baseline rs-fMRI data from 56 patients with TRD subsequently randomized to MBCT or HEP and from 28 healthy control (HC) subjects. This approach was leveraged to test the hypothesis that TRD, relative to HC subjects, involves perturbation of hierarchical gradients among canonical large-scale brain networks (24). To aid with interpreting gradient-based deficits in network topography, we further contextualize the results by using a complementary measure of nodal dysfunction based on network topology, specifically the nodal degree (25).

## METHODS AND MATERIALS

### Subjects

All participants or their surrogates provided written informed consent prior to participation in accordance with the Declaration of Helsinki. The University of California, San Francisco, Committee on Human Research approved the study.

An initial cohort of 59 patients with TRD were enrolled in a randomized controlled behavioral intervention study that included baseline and posttreatment fMRI sessions, and 30 HC subjects were recruited to provide normative baseline fMRI data. Participants were recruited from outpatient psychiatry and general medicine clinics at the University of California, San Francisco, the outpatient psychiatry clinic at Kaiser Permanente in San Francisco, and through advertisements and clinical referrals (26,27). Eligibility screening for TRD was completed in person. Eligible patients met Structured Clinical Interview for DSM-IV-TR Axis I (28) criteria for MDD and had a 17-item Hamilton Depression Rating Scale (HDRS-17) score of 14 or greater. Furthermore, to qualify as having TRD, patients had to be taking antidepressant medication with evidence of 2 or more adequate trials prescribed during the current episode as assessed with the Antidepressant Treatment History Form (29). Patients were excluded for the following: lifetime history of bipolar disorder, schizophrenia, or any psychotic disorder; substance abuse or dependence within 3 months of study onset; being currently suicidal, dangerous to others, or self-injurious; undergoing psychotherapy during the 8-week treatment portion of the study; or a score of <25 on the Mini-Mental State Examination (30).

The HC group was matched to the TRD group on age, gender, and handedness and had no history of a major Axis I psychiatric disorder, neurological illness, or current use of psychotropic medication. Participants were required to be at least 18 years of age, be fluent in English, have no MRI contraindications, and have normal or corrected-to-normal vision.

For each participant, we additionally assessed depressive symptoms through the Quick Inventory of Depression Symptomatology, 16-Item Self-Report (31) and Nolen-Hoeksema’s Response Styles Questionnaire (RSQ22) (32); levels of mindfulness with the Five Facet Mindfulness Questionnaire (FFMQ) (33); and levels of state and trait anxiety through the State-Trait Anxiety Inventory (STAI) (trait and state) (34). Study participants self-reported race and ethnicity, gender, handedness, and years of education.

From the initially recruited sample, 2 HC subjects and 3 patients with TRD had to be excluded based on excessive head movement in the scanner (see details below in Neuroimaging Data Acquisition and Preprocessing), resulting in the final analyzed sample of 56 patients with TRD and 28 HC subjects (Table 1).

### Protocol

Patients with TRD were part of a randomized controlled trial comparing MBCT with an HEP as adjunctive treatments to ongoing antidepressant medication (26,27). Briefly, MBCT involved guided meditations (35); the HEP involved activities to promote health (36). Patients were assessed with rs-fMRI at baseline and following the intervention, while HC subjects were assessed at baseline and did not undergo treatment (26,27). Of the 56 patients with TRD included in our study, 27 went through the MBCT intervention and 29 went through the HEP intervention. Additional details are available in the Supplement and in previously published work. Only rs-fMRI data at baseline were analyzed in the present study.

### Neuroimaging Data Acquisition and Preprocessing

Neuroimaging data were acquired on a Siemens 3T TIM Trio scanner located at the University of California, San Francisco, Neuroimaging Center. A high-resolution anatomical scan was acquired using a 3-dimensional magnetization-prepared rapid acquisition gradient-echo sequence, with a scan time of 5 minutes and 17 seconds, flip angle of 9°, field of view of 220 mm^2^, 160 slices per slab, 1.2-mm thickness, no gap, repetition time of 2.30 seconds, and echo time of 2.94 ms. Functional scans were acquired using a blood oxygen level–dependent echo planar imaging sequence, with a repetition time of 2 seconds, echo time of 30 ms, field of view of 220 mm^2^, flip angle of 77°, bandwidth of 2298 Hx/pixel, 64 × 64 matrix, and 30 slices (3-mm thick, 1-mm gap). Scans were acquired in an axial-oblique plane, parallel to the anterior commissure-posterior commissure line. Participants were instructed to rest with their eyes open during the 5-minute, 24-second blood oxygen level–dependent echo planar imaging functional sequence.

fMRIPrep software (https://fmriprep.org/en/stable/) (37) was used for data preprocessing. Anatomical magnetization-prepared rapid acquisition gradient-echo images were corrected for intensity nonuniformity, skull-stripped, and segmented for cerebrospinal fluid, white matter, and gray matter. Volume-based spatial normalization to Montreal Neurological Institute (MNI) standard space was performed through nonlinear registration of the magnetization-prepared rapid acquisition gradient-echo with the T1-weighted MNI template brain (ICBM152). The first 5 functional volumes were removed to allow for scanner equilibration, resulting in a total number of 157 volumes for the analyses. A mean reference volume and its skull-stripped version were generated and then coregistered to the structural reference using affine registration. Head motion parameters (transformation matrices and the 6 corresponding rotation and translation parameters) were estimated and used to compute framewise head displacement time series. Functional images were slice-time corrected, realigned, and normalized to MNI standard space by applying the structural transformation matrix to the coregistered functional data. The resulting volumes with 2-mm^3^ isotropic resolution were spatially smoothed with a 6-mm radius Gaussian kernel and bandpass filtered in the 0.008 to 0.15 Hz frequency range. Nuisance parameters in the preprocessed data were estimated for the cerebrospinal fluid and white matter. Additional nuisance parameters included the 3 translational and 3 rotational motion parameters, the temporal derivatives of the previous 8 terms (white matter, cerebrospinal fluid, 6 motion time series), and the squares of the previous 16 terms (38,39). Nuisance parameters were filtered for the same frequency range as rs-fMRI data and regressed out from the filtered rs-fMRI data (38,39). The denoised data were used in subsequent analyses. Subjects were included only if their mean framewise head displacement in the scanner (38,39) was below the threshold of 0.55 mm recommended in previous work (40). Global signal regressed rs-fMRI data were also generated using the time series extracted from a whole-brain mask and used for control analyses.

### Functional Connectivity Gradients

**Figure 1.**
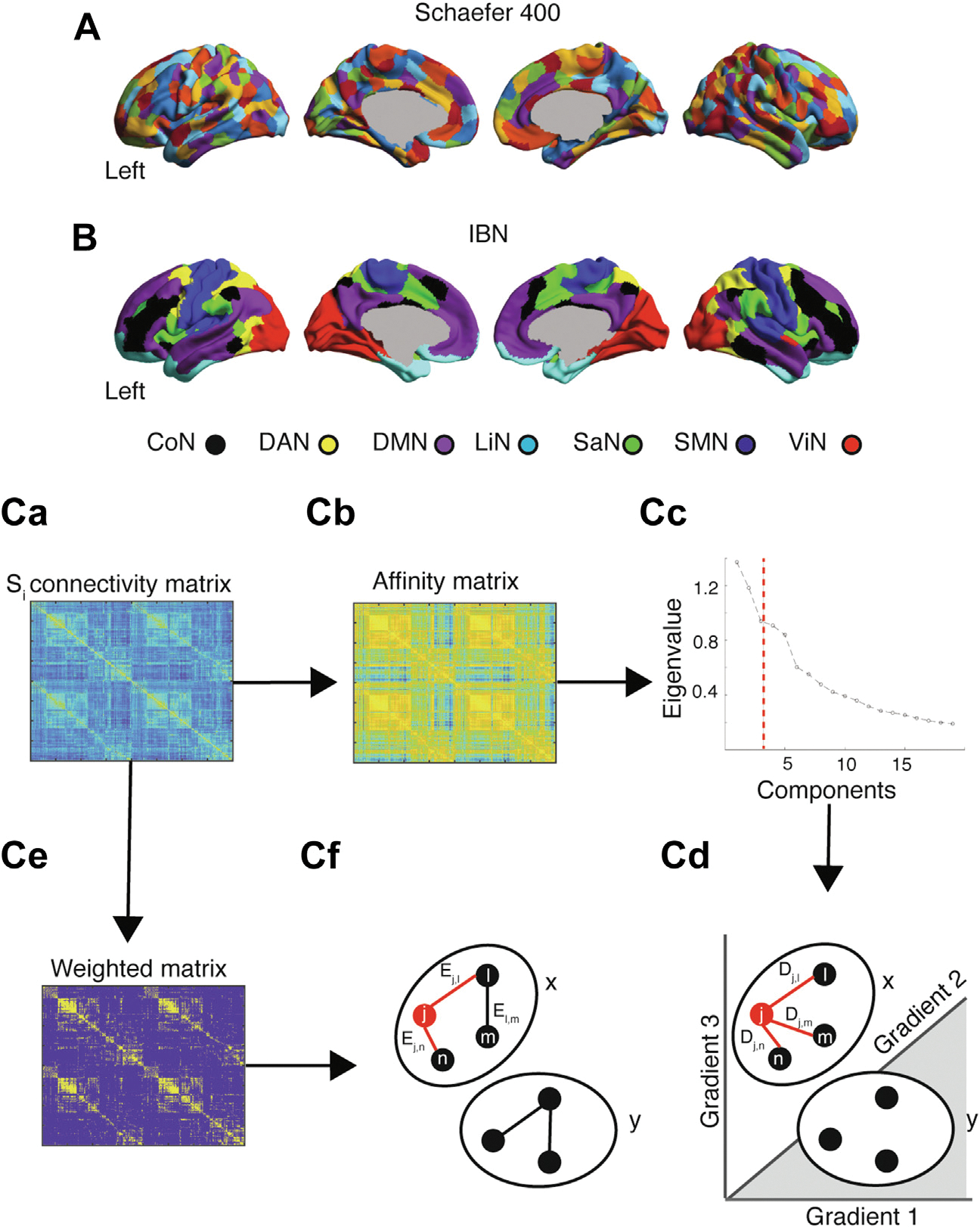
Analytic pipeline. **(A)** 400 nodes from the Schaefer Atlas, each overlapping with **(B)** a specific intrinsic brain network (IBN), were used to derive functional connectivity matrices using resting-state functional magnetic resonance imaging data of healthy control subjects and patients with treatment-resistant depression. **(Ca)** Individual connectivity matrices (S_i_) went through two distinct processing pipelines. To derive cortical connectivity gradients (upper stream), individual connectivity matrices were transformed to **(Cb)** affinity matrices using cosine similarity and **(Cc)** Laplacian decomposition, which was used to derive three primary connectivity gradients. Combined, these gradients explained 34.9% of the variance in functional connectivity (red dashed line). **(Cd)** The position of an individual node belonging to a specific intrinsic brain network (e.g., Network x) was used to derive a topographical measure of nodal dispersion, reflecting the average Euclidean distance (D) in gradient space between a node and all other nodes belonging to the same network. Individual connectivity matrices were also leveraged to derive topological measures of nodal degree (lower stream). **(Ce)** Connectivity matrices were weighted by binarizing at a connectivity threshold of 0.35. **(Cf)** For each node within a network, we assessed the level of degree by counting the edges (E) of this node to all other nodes within a network and dividing by the total amount of edges. CoN, control network; DAN, dorsal attention network; DMN, default mode network; LiN, limbic network; SaN, salience network; SMN, sensorimotor network; ViN, visual network.

The Schaefer Atlas (41) was used to derive rs-fMRI activity time series for 400 cortical regions (Figure 1A, B). The Pearson correlation was applied to the regional activity time series to derive individual functional connectivity matrices (Figure 1C) and group mean functional connectivity matrices for HC subjects and patients with TRD (Figure S1).

The diffusion embedding approach (17,18), as implemented by the BrainSpace toolbox (https://brainspace.readthedocs.io/en/latest/pages/getting_started.html) (23), was then applied to the HC group mean functional connectivity matrix to estimate connectivity gradients. Briefly, the top 10% strongest functional connections were retained for each parcel, referred to hereafter as a node, and cosine similarity was calculated between each pair of nodes to generate a dissimilarity matrix (Figure 1C) (42,43). Diffusion map embedding was then applied to decompose the functional connectome into primary components, referred to as gradients, with each gradient explaining varying levels of variance in connectivity (Figure 1C). These gradients discriminate across levels of the cortical hierarchy (i.e., sensory processing vs. higher-order cognition), whereas node-specific gradient values reflect the similarity in connectivity along this sensory-transmodal axis. An identical approach was used to derive connectivity gradients from the TRD group mean connectivity matrix and from the connectivity matrices of individual participants. The resulting gradient maps were subsequently aligned to the gradients derived at the group level in HC subjects using iterative Procrustes rotation, therefore enabling comparisons across individual embedding solutions (20,23,44). Control analyses were performed with publicly available cortical gradients maps (see the Supplement) (17).

### Nodal Dispersion

For each participant, we then derived a measure of within-network nodal dispersion. We plotted the first 3 connectivity gradients—because these explained most of the underlying variance (see elbow plot in Figure 1C)—against each other to derive a 3-dimensional manifold in which we calculated the Euclidean distance between nodes belonging to the same intrinsic brain network (Figure 1D) (44). Nodal dispersion was derived for each node belonging to a specific intrinsic brain network and averaged across nodes within intrinsic brain networks, yielding a final estimate of within-network nodal dispersion for each participant. We performed several control analyses to assess the impact of methodological parameters on our analyses (see Supplement). Further, we derived a measure of between-network nodal dispersion calculated as the Euclidean distance between network centroids (i.e., the arithmetic mean in gradient space of all nodes belonging to the same network).

### Nodal Degree

In parallel with the connectivity gradient approach, we also derived a traditional measure of within-network nodal degree for all participants (25) by using the publicly available Brain Connectivity Toolbox (https://sites.google.com/site/bctnet/). Nodal degree is a widely used measure of network topology commonly derived using graph-theoretical approaches (25). Briefly, individual connectivity matrices were thresholded for correlation values below 0.35 (retaining a median of 26% of connections) and binarized (Figure 1D). To control for threshold choice, measures of nodal degree were derived also for connectivity thresholds of 0.45 and 0.25 (retaining 16% and 38% of connections, respectively). At any threshold, patients with TRD and HC subjects did not significantly differ in respect to the density of retained connections. Weighted connectivity matrices were used to count the number of surviving edges between a specific node within a network and all other nodes within the same network (Figure 1D). The sum of surviving edges for a node was then divided by the total amount of edges within the network. Nodal degree measures were derived for each single node in a network and averaged across nodes in the same network. This procedure resulted in a measure of within-network nodal degree reflecting the level of integration between nodes belonging to the same network.

### Statistical Analyses

In-house MATLAB R2021a (The MathWorks, Inc.) and R 4.1.1 (R Foundation for Statistical Computing) scripts were used to perform the statistical analyses. See the Supplemental Methods for more details.

## RESULTS

### Cortical Connectivity Gradients in HC Subjects and Patients With TRD

**Figure 2.**
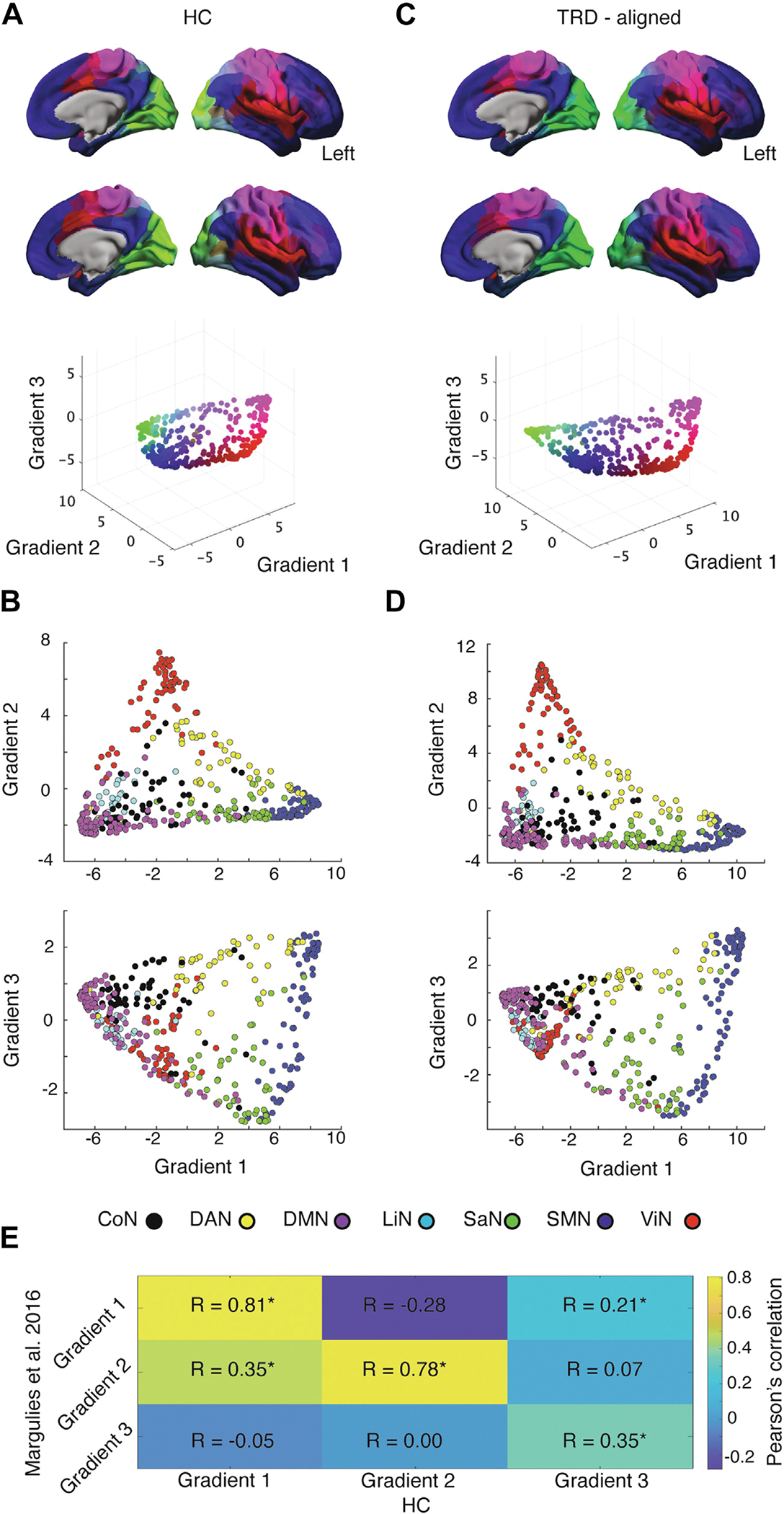
Cortical connectivity gradients. **(A)** Cortical connectivity gradients of healthy control (HC) subjects projected into cortical surface. The 3-dimensional scatterplot below shows how individual nodes distributed along the first 3 gradients. Colors reflect the loadings of nodes on individual gradients. For example, the sensorimotor cortex appears purple and regions overlapping with the default mode network (DMN) appear blue, reflecting that these systems respectively anchor the extremes of gradient 1. **(B)** Scatterplots reflecting how nodes belonging to distinct intrinsic brain networks align along cortical gradients in HC subjects. **(C)** Spatial correlation between maps of gradients 1–3 in HC subjects and maps of gradients 1–3 using publicly available maps of canonical cortical gradients. **(D)** Cortical connectivity gradients of patients with treatment-resistant depression (TRD) aligned to the gradients of HC subjects following Procrustes rotation. **(E)** Scatterplots reflecting how nodes belonging to distinct intrinsic brain networks align along cortical gradients in patients with TRD. *p < .005. CoN, control network; DAN, dorsal attention network; LiN, limbic network; SaN, salience network; SMN, sensorimotor network; ViN, visual network.

We applied a diffusion gradient approach separately on rs-fMRI–based connectivity data from HC subjects and patients with TRD to derive cortical connectivity gradients reflecting processing hierarchies spanning sensory and transmodal areas (Figure 2; Figure S2A). The first 3 principal gradients derived from rs-fMRI data of HC subjects explained 34.9% of the variance in functional connectivity (elbow plot in Figure 1C). Gradient 1 anchored sensorimotor areas at its positive extreme, while regions belonging to the DMN were located at the opposite, negative extreme (Figure 2A, B). Sensorimotor and DMN areas occupied the negative extreme on gradient 2, while visual-sensory areas populated the positive end of this gradient (Figure 2A, B). Notably, these first 2 connectivity gradients overlap with previously reported gradients in functional connectivity, structural connectivity, myelin density, and genetic expression (17,18), which consistently separate sensory processing regions from transmodal areas of the DMN. Gradient 3 showed a more complex pattern, segregating regions of the dorsal attention network from regions belonging to the salience network, potentially reflecting a higher-order, attention-related gradient separating regions attending to externally presented cues (45) from regions devoted to processing visceral and interoceptive information (46,47). The normative gradients identified in our HC subject sample showed strong to moderate correspondence to gradients described in prior foundational work (Figure 2C) (17). Similar fundamental properties of hierarchical brain organization were found in patients with TRD after aligning the principal connectivity gradients of patients to those of HC subjects (Figure 2D, E), in support of the notion that cortical gradients reflect fundamental properties of brain topography in both health and disease (17,18,20,21). Gradients 4 to 6 explained a lower amount of variance and showed less discernible patterns of regional variation (Figure S2).

### Within-Network Nodal Dispersion

**Figure 3.**
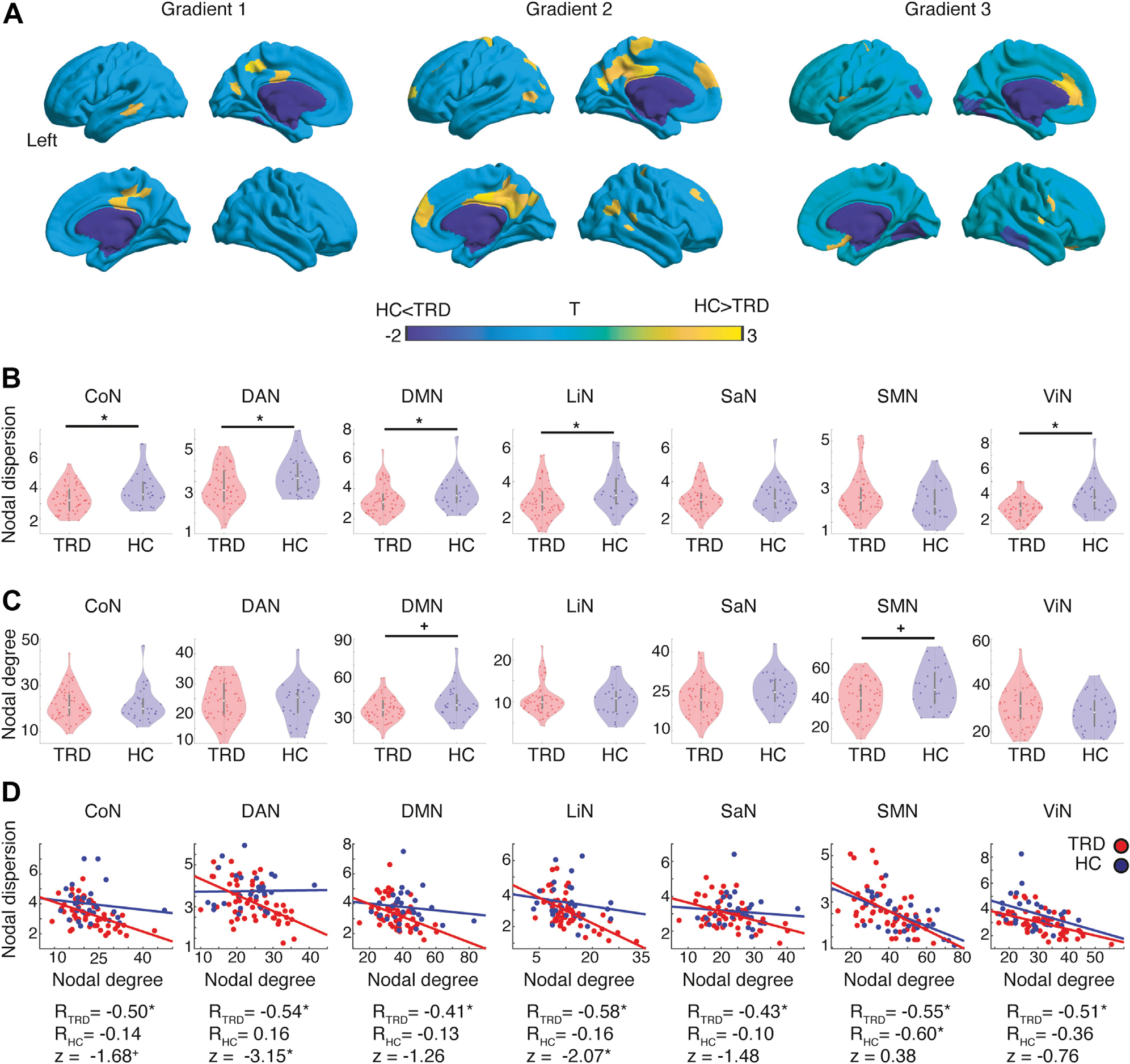
Nodal dispersion and nodal degree. **(A)** Nodewise statistical comparisons between healthy control (HC) subjects and patients with treatment-resistant depression (TRD), with increases/decreases in the TRD group shown in cold/warm colors (*p* < .05 uncorrected). **(B)** Violin plots reflecting topographical differences in within-network nodal dispersion between patients with TRD (red) and HC subjects (blue). **(C)** Violin plots reflecting topological differences in within-network nodal degree between patients with TRD and HC subjects. **(D)** Scatterplots reflecting the association between within-network nodal degree and within-network nodal dispersion separately for patients with TRD and HC subjects. Pearson correlation coefficients are reported below the scatterplots for each group separately, together with associated Fisher *r*-to-*z* tests for independent samples comparing the strength of the correlations across groups. *p < .05, false discovery rate corrected, +*p* < .05, uncorrected. CoN, control network; DAN, dorsal attention network; DMN, default mode network; LiN, limbic network; SaN, salience network; SMN, sensorimotor network; ViN, visual network.

Node-level gradient comparisons (*p* < .05, uncorrected) revealed increased gradient scores in patients with TRD in sensory and early transmodal regions, such as the ventromedial occipital and posterior inferior temporal cortices, together with decreased gradient scores in transmodal areas including the precuneus and the medial prefrontal and cingulate cortices (Figure 3A). We then derived a measure of within-network nodal dispersion (Figure 1D), reflecting the level of connectedness of nodes belonging to the same intrinsic brain network (44). A 2-way analysis of variance revealed a main effect of network (*F*_6,567_ = 15.2, *p* < .0005) and a main effect of group (*F*_2,567_ = 18.0, *p* < .0005). Pairwise comparisons revealed that all networks, except for the salience and sensorimotor networks, showed reduced within-network nodal dispersion in patients with TRD compared with HC subjects (*p* < .05, false discovery rate corrected for multiple comparisons) (Figure 3B), suggesting overall higher within-network connectedness. We performed control analyses to assess the impact of head movement on within-network dispersion and assessed the impact of methodological parameters including 1) global signal regression, 2) atlas parcellation, 3) gradient decomposition through Laplacian embedding, 4) angular normalization to generate the dissimilarity matrices, 5) adding gradients 4 to 6 when computing within-network nodal dispersion, or 6) using publicly available gradient maps to derive individual gradients (see Supplemental Results, Figures S2–S4, and Tables S1 and S2).

**Figure 4.**
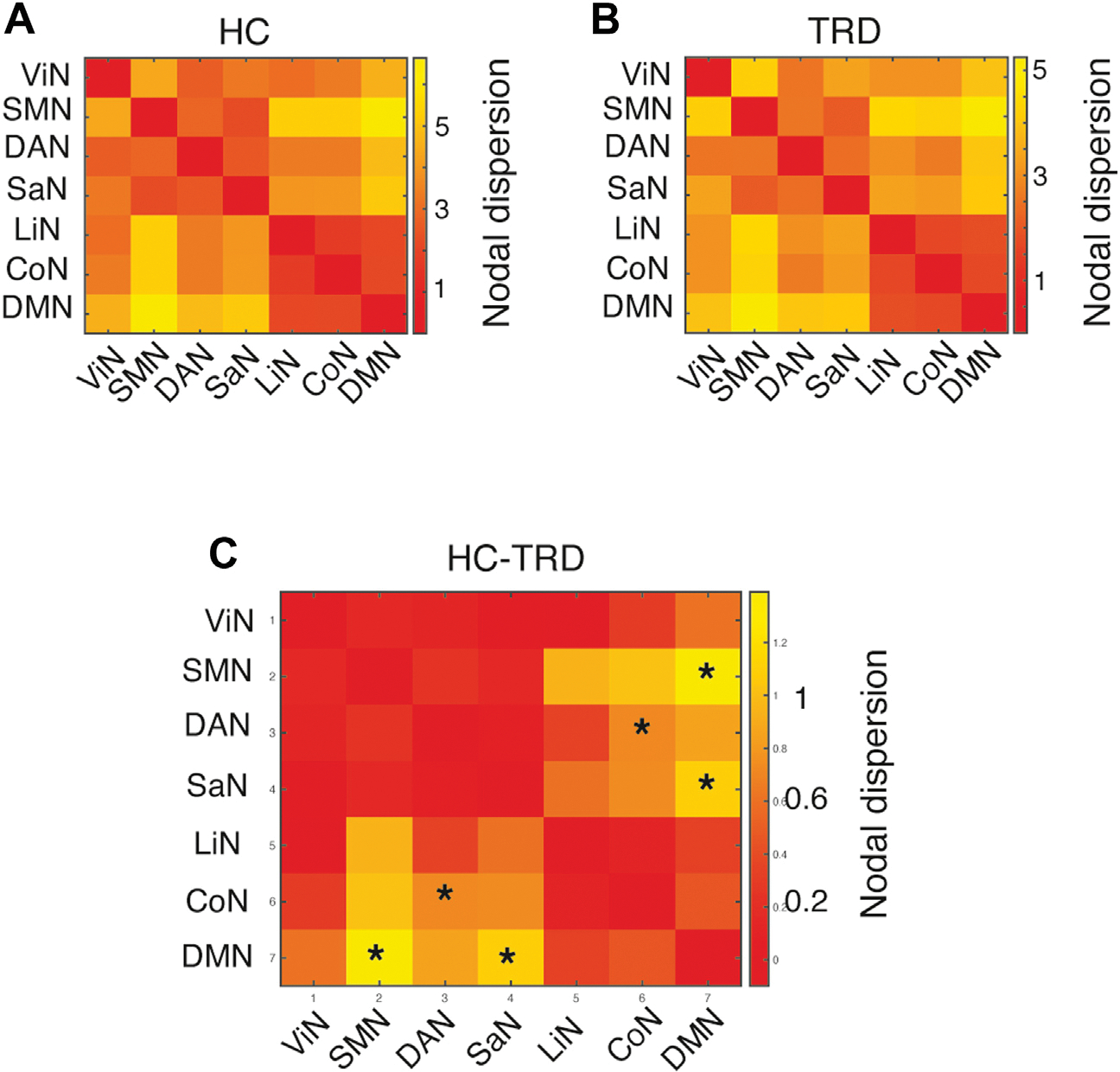
Between-network nodal dispersion. Between-network nodal distance in **(A)** healthy control (HC) subjects and (B) patients with treatment-resistant depression (TRD). **(C)** Significant reductions in between-network nodal dispersion were found in patients with TRD, affecting the sensorimotor network (SMN) and default mode network (DMN), the salience network (SaN) and DMN, and the control network (CoN) and dorsal attention network (DAN). None of these findings survived false discovery rate correction for multiple comparisons. *p < .05, uncorrected. LiN, limbic network; ViN, visual network.

We analyzed whether TRD also affected cortical hierarchies between networks in addition to within-network gradient organization. We derived a measure of between-network nodal dispersion that revealed reduced nodal dispersion in patients with TRD between the sensorimotor network and DMN, between the salience network and DMN, and between the CoN and dorsal attention network, although none of these findings survived correction for multiple comparisons (*p* < .05, uncorrected) (Figure 4).

### Within-Network Nodal Degree

Comprehensively, the previous findings suggest that in TRD, nodes belonging to the same network are more integrated to each other. To confirm this hypothesis, we derived a complementary measure of nodal integration based on graph theoretical approaches, namely within-network nodal degree. A 2-way analysis of variance revealed a main effect of network (*F*_6,567_ = 187.9, *p* < .0005) and a weaker main effect of group (*F*_2,567_ = 3.1, *p* < .05). Pairwise comparisons revealed that there were no significant between-group differences in within-network degree that survived multiple comparisons. However, the DMN and sensorimotor network nodal degree was significantly lower in patients with TRD compared with HC subjects (*p* < .05, uncorrected) (Figure 3C).

When relating within-network nodal dispersion to within-network nodal degree, we consistently found a significant negative association between both measures, particularly in patients with TRD and to a lesser extent in HC subjects (*p* < .05, false discovery rate corrected for multiple comparisons if not reported otherwise, Pearson correlation coefficients and associated Fisher *r*-to-*z* tests for independent samples comparing the strength of correlations across groups reported in the plots) (Figure 3D). Notably, these findings were robust across distinct thresholds applied to generate the weighted connectivity matrices used to estimate nodal degree (Figure S3). In summary, these findings support the notion that decreased within-nodal dispersion, at least in patients with TRD, reflects within-network hyperconnectedness. This negative association between nodal measures was prominent in patients with TRD but not as prominent in HC subjects, suggesting a more complex relationship between cortical topology and topography in the healthy human brain.

### Within-Network Nodal Dispersion and Baseline Symptoms of Depression

**Figure 5.**
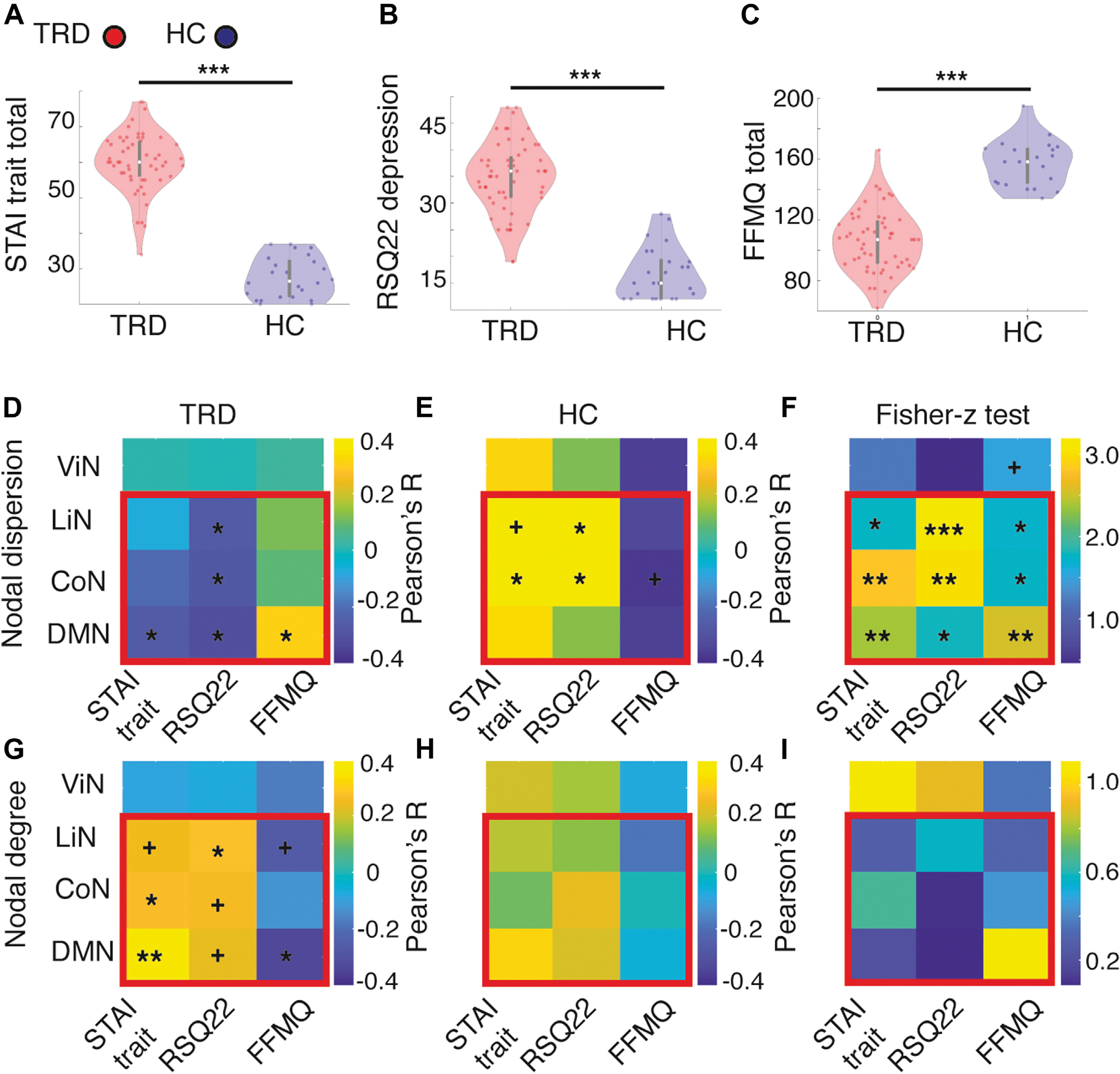
Nodal dispersion correlates with symptoms of depression. **(A)** Levels of trait anxiety (State-Trait Anxiety Inventory [STAI] trait total scores) and **(B)** depression (Nolen-Hoeksema’s Response Styles Questionnaire [RSQ22]) were significantly higher in patients with treatment-resistant depression (TRD) (red violin plots) when compared with healthy control (HC) subjects (blue violin plots), while levels of **(C)** mindfulness (Five Facet Mindfulness Questionnaire [FFMQ] total scores) were significantly lower in patients with TRD when compared with HC subjects. **(D, E)** Within-network nodal dispersion of the default mode network (DMN), control network (CoN), and limbic network (LiN) correlated negatively with trait anxiety and depression and positively with mindfulness in patients with TRD but not in HC subjects. No significant correlations were found for dispersion of the visual network (ViN), suggesting a specific association of clinical measures to higher-order cognitive and limbic networks. The matrix in panel **(F)** reflects Fisher *r*-to-*z* tests for independent samples comparing the strength of the correlations across groups. **(G, H)** Conversely, the within-network nodal degree of the DMN, CoN, and LiN correlated positively with trait anxiety and depression and negatively with mindfulness in patients with TRD but not in HC subjects. The matrix in panel **(I)** reflects Fisher *r*-to-*z* tests for independent samples comparing the strength of the correlations across groups. ^+^*p* < .1, *p < .05, **p < .005, ***p < .0005.

Given the recurrent association of the DMN, CoN, and LiN with clinical symptoms of depression (9,15,16), we first investigated the association of within-network nodal dispersion and degree in these systems with clinical depression severity in patients as assessed with the HDRS-17. Within-network nodal dispersion of any network did not significantly correlate with HDRS-17, although within-network nodal degree of the CoN and LiN positively correlated with HDRS scores (Table S3). Subsequently, we assessed the relationship between within-network nodal dispersion of the DMN, CoN, and LiN and clinical measures of increased anxiety, depressed mood, and reduced mindfulness (16,26,27). To assess whether associations between nodal dispersion and clinical measures were specific to higher-order cognitive and emotional systems, we also report correlations between clinical measures and nodal dispersion of the visual network. In line with previous work, our patient sample showed increased levels of trait anxiety as measured through the STAI questionnaire (*p* < .0005) (Figure 5A), increased levels of depressive symptoms using the RSQ22 (*p* < .0005) (Figure 5B), and decreased levels of mindfulness (26,27) as measured through the FFMQ (*p* < .0005) (Figure 5C). Within-network nodal dispersion of the DMN, CoN, and LiN negatively correlated with trait anxiety and depression, while it positively correlated with mindfulness in patients with TRD but not in HC subjects (Figure 5D, E). Dispersion of the visual network did not significantly correlate with any clinical measure. Consistent with the previously described negative relationship between nodal dispersion and nodal degree, within-network nodal degree of the DMN, CoN, and LiN positively correlated with trait anxiety and depression, while it negatively correlated with mindfulness in patients with TRD but not in HC subjects (Figure 5G, I).

### Within-Network Nodal Dispersion and Change Scores in Clinical Symptoms

**Figure 6.**
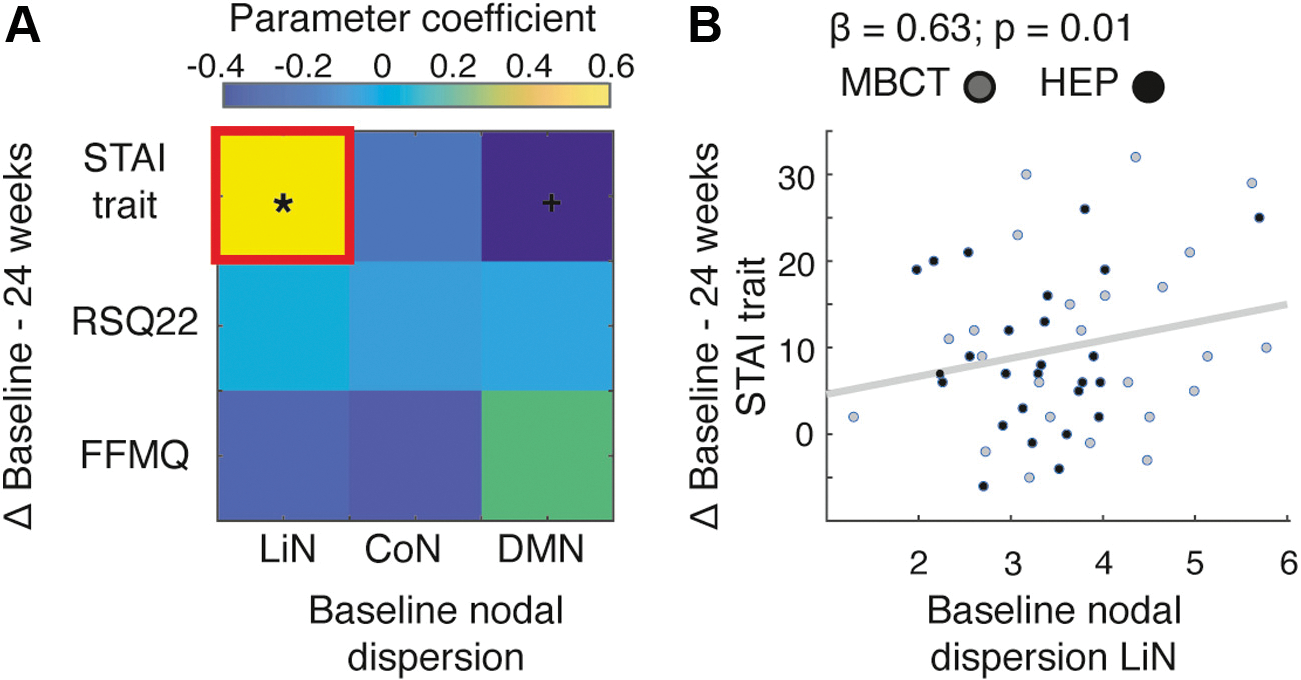
Baseline limbic network (LiN) nodal dispersion predicts change in State-Trait Anxiety Inventory (STAI) trait scores following a mindfulness-based cognitive therapy (MBCT)/health enhancement program (HEP) intervention. **(A)** Parameter regression coefficients from multiple regression models predicting clinical score changes (difference of baseline minus 24 weeks) from baseline within-network nodal dispersion. +*p* < .01, *p < .05. **(B)** Only nodal dispersion of the LiN significantly predicted STAI trait change scores. CoN, control network; DMN, default mode network; FFMQ, Five Facet Mindfulness Questionnaire; RSQ22, Nolen-Hoeksema’s Response Styles Questionnaire.

In line with our previous studies (26,27), patients with TRD on the MBCT arm showed greater HDRS-17 reductions relative to the control intervention, although in our study the effect was not significant (*F*_1,107_ = 3.07, *p* = .08) (Figure S5) (26,27), likely due to the smaller patient subset in this sample following head movement control. We then assessed whether within-network nodal dispersion at baseline could predict STAI trait, FFMQ, and RSQ22 change scores, as these clinical questionnaires correlated with baseline nodal dispersion. A repeated measurement analysis of variance revealed a main effect of time (but no effect of group), with improved STAI trait, FFMQ, and RSQ22 scores after 8 and 24 weeks in both the HEP and MBCT arms (Figure S6 and Table S4). Multiple regression analyses revealed that LiN nodal dispersion at baseline predicted STAI trait change scores 24 weeks after the intervention (β_1,46_ = 0.63, *p* = .01) (Figure 6).

## DISCUSSION

Functional connectivity of the human cortex can be decomposed into primary gradients that anchor on one end primary sensory and motor areas, and on the other end transmodal regions overlapping with the DMN. This study explored how TRD impacts this fundamental topography of hierarchical cortical organization. We capitalized on rs-fMRI data acquired in patients with TRD and HC subjects and applied recently developed gradient extraction tools to assess gradient imbalances within major intrinsic brain networks. Although the global hierarchical architecture was similar across the 2 groups, we found that brain regions belonging to the same network were located more closely to each other in topographical gradient space in patients with TRD relative to HC subjects. Reduced within-network nodal dispersion correlated with higher levels of nodal degree derived through graph theory–based topology measures, overall suggesting higher within-network functional integration in TRD. In the patient group, decreased nodal dispersion of higher-order cognitive and limbic networks correlated with depression, anxiety, and reduced mindfulness at baseline. Change in anxiety scores following a mindfulness-based intervention were predicted by limbic nodal dispersion. Overall, these findings suggest deleterious cortical network topography and topology in TRD and underscore the role of higher-order and limbic networks in mediating core symptoms of depression.

### Increased Within-Network Integration in TRD

The pervasive correlation between nodal degree and nodal dispersion in our patient sample suggests that TRD impacts cortical hierarchies by driving hyperintegration within several brain networks (48). Other neuropsychiatric conditions have been shown to impact cortical connectivity gradients. Autism spectrum disorder has been shown to alter brain topography by showing atypical connectivity transitions between sensory and higher-order DMN regions (20). Our findings align with previous reports of altered cortical gradient organization in individuals with cognitive vulnerability to depression (22) and in a larger sample of patients with MDD (21). Individuals with cognitive vulnerability to depression have been shown to display reduced gradient scores in the left insula, which correlated with lower attentional scores in patients, suggesting that gradient reorganization may precede the onset of depression (22). A recent study involving a large sample of patients showed that MDD exhibits abnormal global topography of the principal sensory-to-transmodal gradient (21). These focal alterations of gradient scores mostly affected transmodal areas implicated in higher-order cognition overlapping with the DMN (21).

### Brain Network Hyperintegration Mediates Symptoms of Depression

Despite numerous efforts to map brain network dysfunctions in depression, important inconsistencies exist regarding the location and directionality of connectivity changes, with both hyperconnectivity (15) and hypoconnectivity (49) findings reported in the literature. Disease duration, perseverance of symptoms, and heterogeneous subtypes of depression (8,9) may account for important sources of variability, as do head movement in the scanner and differing data acquisition protocols and preprocessing pipelines (38–40). Although our findings contrast with reports of decreased connectivity in attentional networks (10), they align well with previous reports of DMN hyperconnectivity found in patients with depression (9,15). Hyperconnectivity among DMN regions in depression is consistent with our interpretation of reduced nodal dispersion reflecting within-network hyperintegration. Prior studies in both HC subjects and patients with depression have associated DMN hypersynchrony with self-referential processes affected in depression, including reduced mindfulness and social-emotional dysfunctions (15,16,50), suggesting a deleterious nature of DMN hyperintegration in TRD.

### Limitations and Future Directions

Three limitations need to be considered when interpreting our findings as potential evidence of within-network hyperintegration in TRD. First, methods used to extract connectivity gradients may need further refinements when addressing gradient changes at the individual level and across clinical populations. Although findings of reduced within-network nodal dispersion were consistently found when using global signal regression or medium- to high-parcellated atlases, the method chosen to derive cortical connectivity gradients greatly influenced the analyses. Second, nodal dispersion in patients with TRD neither correlated with the HDRS-17 nor predicted clinical improvement following either MBCT or HEP (except for the LiN). Gradient approaches have been mostly applied to study fundamental aspects of brain functioning by leveraging large samples. Our analyses may have experienced sample size issues affecting both patients and control subjects. Given the recent discovery of distinct biotypes in MDD (8,9), longitudinal studies involving larger patient samples are needed to validate our findings. Future studies should confirm whether decreased nodal dispersion is a generalizable marker of network hyperintegration in TRD and whether nodal dispersion can be normalized following tailored behavioral and pharmacological interventions.

## Supplementary Material

supplement

## Figures and Tables

**Table 1.** Participant Demographic and Clinical Characteristics at Baseline

	HC Group, *n* = 28	TRD Group, *n* = 56	Statistic	*p*
Age, Years	45.4 (9.3)	42.9 (9.9)	*t*_82_ = 1.14	.268
Female	20	44	*x*^2^_1_ = 0.21	.651
Handedness, Ambidextrous/Left/Right	1/2/25	2/5/49	*x*^2^_2_ = 0.08	.962
Education, Years	16.9 (2.5)	16.1 (2.1)	*t*_81_ = 1.57	.101
Hispanic-Latino	4	4	*x*^2^_1_ = 0.40	.529
Asian/Black/Other/White	1/2/0/25	6/4/1/45	*x*^2^_3_ = 12.38	<.01
FD, mm	0.23 (0.10)	0.25 (0.11)	*t*_82_ = −1.01	.343
Spike Occurrence, Number of Volumes With FD >0.5 mm	7.5 (14.4)	13.4 (18.9)	*t*_82_ = 1.45	.149
Age of MDE Onset, Years	-	20.8 (10.1)	-	-
Number of MDEs	-	3.6 (2.5)	-	-
Current Onset Duration, Months	-	85.6 (110.5)	-	-
Number of Trials	-	2.9 (1.3)	-	-
Concurrent Medication at Baseline				
Antidepressants	-	56 (100.0%)	-	-
Mood stabilizers	-	8 (14.3%)	-	-
Sedatives	-	19 (33.9%)		
Stimulants	-	13 (23.2%)	-	-
Antipsychotics	-	1 (1.8%)	-	-
Other	-	1 (1.8%)	-	-
Clinical Questionnaire Scores				
HDRS-17	1.6 (1.3)	17.4 (2.7)	*t*_82_ = −35.5	<.001
QIDS-SR16	2.6 (1.4)	14.9 (3.7)	*t*_79_ = −21.6	<.001
STAI trait	27.6 (5.8)	60.1 (8.5)	*t*_76_ = −19.6	<.001
STAI state	26.5 (7.8)	56.3 (9.8)	*t*_78_ = −14.5	<.001
RSQ22	31.8 (9.0)	59.7 (11.0)	*t*_78_ = −12.0	<.001
FFMQ	157.2 (15.2)	106.1 (20.0)	*t*_74_ = 12.0	<.001

Values are mean (SD), n, or n (%).

FD, framewise head displacement; FFMQ, Five Facet Mindfulness Questionnaire; HC, healthy control; HDRS-17, 17-item Hamilton Depression Rating Scale; MDE, major depressive episode; QIDS-SR16, Quick Inventory of Depression Symptomatology, 16-Item Self-Report; RSQ22, Nolen-Hoeksema’s Response Styles Questionnaire; STAI, State-Trait Anxiety Inventory; TRD, treatment-resistant depression.
