# Supplementary material for: Dysfunctional Cortical Gradient Topography in Treatment-Resistant Major Depressive Disorder
Source: Biol Psychiatry Cogn Neurosci Neuroimaging. Author manuscript; Available in PMC 2023 Sep 28. (PMC10150583; doi:10.1016/j.bpsc.2022.10.009)
Supplement: supplement [file NIHMS1889634-supplement-supplement.docx]

# **SUPPLEMENTARY INFORMATION**

**Dysfunctional Cortical Gradient Architecture in**

**Treatment-Resistant Major Depression**

Pasquini *et al.*

**Content:**

1. Supplementary Methods:

- Protocol

- Schaefer Atlas

- Nodal dispersion

- Statistical analyses

2. Supplementary Results:

- Control analyses

3. Supplementary Figures and Tables:

- Six Supplementary Figures and four Supplementary Tables

4. Supplementary References

**Supplementary Methods**

## *Protocol*

TRD patients were part of a randomized controlled trial comparing MBCT to a HEP as adjunctive treatments to ongoing antidepressant medication. From our final sample of 56 TRD patients, 27 underwent MBCT and 29 underwent HEP. Details regarding treatment programs and randomization procedures were described previously (1,2). Briefly, MBCT involved guided meditations and exercises intended to help participants identify cognitive distortions, disengage from rumination, and use nonjudgmental present-moment awareness (3). HEP involved physical exercise, functional movement, music therapy, diet education, and guided imagery intended to promote health and improve mood (4). Both treatment groups met for eight weeks in groups of 6–12 once a week for 135 min. Patients were assessed with rs-fMRI at baseline and following intervention, while HC were assessed at baseline and did not undergo treatment. Only baseline rs-fMRI data from TRD and HC participants are analyzed in the present study. TRD patients underwent clinical assessments at baseline and at weeks 8, 24, 36, and 52 (1,2).

*Schaefer Atlas*

The Schaefer Atlas (5) was used to derive rs-fMRI activity time series for 400 cortical regions (Figure 1A). This data-driven atlas, derived from 1498 healthy individuals, exploits local gradients in functional connectivity, while maximizing the similarity of rs-fMRI time courses within a parcel. One strength of this atlas is that the resulting cerebral cortex parcellations are functionally and connectionally homogeneous and display a one-to-one correspondence to major intrinsic brain networks (6) (Figure 1B). Pearson’s correlation was applied to the regional activity time series to derive individual functional connectivity matrices (Figure 1Ca) and group-mean functional connectivity matrices for HC and TRD participants (Figure S1).

*Nodal dispersion*

We derived a measure of within-network nodal dispersion for each participant by plotting the first three connectivity gradients against each other to derive a topographical three-dimensional Euclidean space. Within this manifold we then calculated the Euclidean distance between nodes belonging to the same intrinsic brain network (7) (Figure 1Cd). More formally, nodal dispersion was defined for each node as:

$$\mathrm{NDj}=(\sum_{j,i}^{n-1} \delta j,i)/(n-1)$$

where NDj refers to the nodal dispersion of an individual node within a specific intrinsic brain network, e.g., the DMN, and n refers to the total number of nodes within this network. δj,i reflects de Euclidean distance between node j and another node i belonging to that network. δj,i is iteratively generated between a node and all other nodes in a network and eventually averaged. Nodal dispersion measures were derived for each node belonging to a specific intrinsic brain network and averaged across nodes, yielding a final estimate of within-network nodal dispersion for each participant. We performed several control analyses to assess the impact of methodological parameters on our findings. We derived measures of within-network nodal dispersion by using: (i) the first six gradients instead of three; (ii) global signal regression on rs-fMRI data; (iii) the Schaefer Altas with 1000 or 200 parcels; (iv) Laplacian embedding instead of diffusion embedding to derive cortical gradients; (v) angular normalization instead of cosine similarity to derive the dissimilarity matrices; (vi) individually decomposed gradient maps projected into canonical cortical gradients derived from a seminal previous study (8). Canonical maps were downloaded from the public repository NeuroVault (<https://neurovault.org/>) (<https://identifiers.org/neurovault.collection:1598>) and parcellated into the Schaefer 400 atlas. Findings from these analyses are presented in the Supplementary Figures and Tables. Finally, we also derived a measure of between-network nodal dispersion calculated as the Euclidean distance between network centroids (i.e., the arithmetic mean of all nodes belonging to the same network).

*Statistical analyses*

Pearson’s correlation was used to assess the spatial similarity between cortical gradients extracted in HCs and the previously introduced canonical cortical gradients derived from a seminal gradient study (8) (<https://identifiers.org/neurovault.collection:1598>), which were parcellated into the Schaefer 400 atlas. Chi-square tests were used to compare sex, handedness, ethnicity, and race distributions across groups (p<0.05 uncorrected). Two sample t-tests were used to compare continuous demographical and clinical variables, functional connectivity matrices, and parcel-level gradient maps between HCs and patients (p<0.05 uncorrected). ANOVA models and associated post-hoc t-test were used to compare measures of within-network nodal dispersion and nodal degree across HCs and patients with TRD (p<0.05 FDR corrected for multiple comparisons if not specified otherwise). Multiple linear regression analyses were used to associate measures of within-network nodal dispersion to age, sex, and number of head movement spikes while scanned, defined as the number of volumes with a head framewise displacement higher than 0.5 mm (p<0.05 uncorrected) (9). Pearson’s correlation analyses were used to associate measures of within-network nodal degree to measures of within-network nodal dispersion separately for each group (p<0.05 FDR corrected for multiple comparisons if not specified otherwise). To compare the strength of the correlations across groups, Fisher r-to-z transformation tests for independent samples were performed using an online calculator (<https://www.psychometrica.de/correlation.html>). Pearson’s correlation analyses were used to associate measures of within-network nodal degree and dispersion to clinical scores at baseline separately for each group (p<0.05 FDR corrected for multiple comparisons if not specified otherwise). Again, Fisher r-to-z tests for independent samples were performed to compare the strength of the correlations across groups. Four repeated measures ANOVA were performed to evaluate the effect of MBCT and HEP interventions over time on HDRS-17 (comparing baseline versus 8 weeks), STAI trait, RSQ22, and FFMQ scores (comparing baseline, 8 weeks, and 24 weeks) of patients with TRD. Multiple linear regression analyses were used to associate within-network nodal dispersion to change in STAI trait, RSQ22, and FFMQ scores at 24 weeks after completing the interventions. Both intervention arms were combined into one sample for this last set of analyses assessing the association between longitudinal clinical change scores and dispersion measures at baseline, since the repeated measures ANOVAs revealed a main effect of time but no effect of group.

**Supplementary Results**

*Control analyses*

We explored the relationship between within-network nodal dispersion in TRD patients and confounds such as number of head movement-related spikes during scanning and demographic variables, including age and sex, using multiple linear regression models (Table S1; p<0.05, uncorrected). These analyses revealed no significant relationships except for a trending association between Visual Network nodal dispersion and head movement spikes. Gradients 4-6 affected group differences when computing measures of within-network nodal dispersion, with only the Visual Network and CoN showing significantly reduced within-network nodal dispersion in TRD (Table S2; p<0.05, FDR corrected for multiple comparisons). We performed additional control analyses to assess the impact of methodological parameters on group differences in within-network nodal dispersion. We found similar cortical gradients as those reported in the main findings regardless of whether we used: (i) global signal regression; (ii) higher or lower parcellated atlases; (iv) gradient decomposition through Laplacian embedding; (v) or angular normalization to generate the dissimilarity matrices (Figure S2 C-F). While data preprocessed with global signal regression or higher atlas parcellation consistently revealed decreased within-network nodal dispersion in TRD, group differences were affected when using a lower parcellated atlas, Laplacian embedding, or angular normalization (Table S2). Measures of nodal dispersion derived by projecting individually decomposed functional connectivity matrices into normative gradient maps derived from Margulies et al. 2016 showed significantly lower dispersion measures in TRD (Figure S4). These findings are analogous to the findings presented in the main manuscript (Figure 3A), which used the group-averaged functional connectivity matrix of the HCs to extract normative gradient maps.

**Supplementary Figures and Tables**


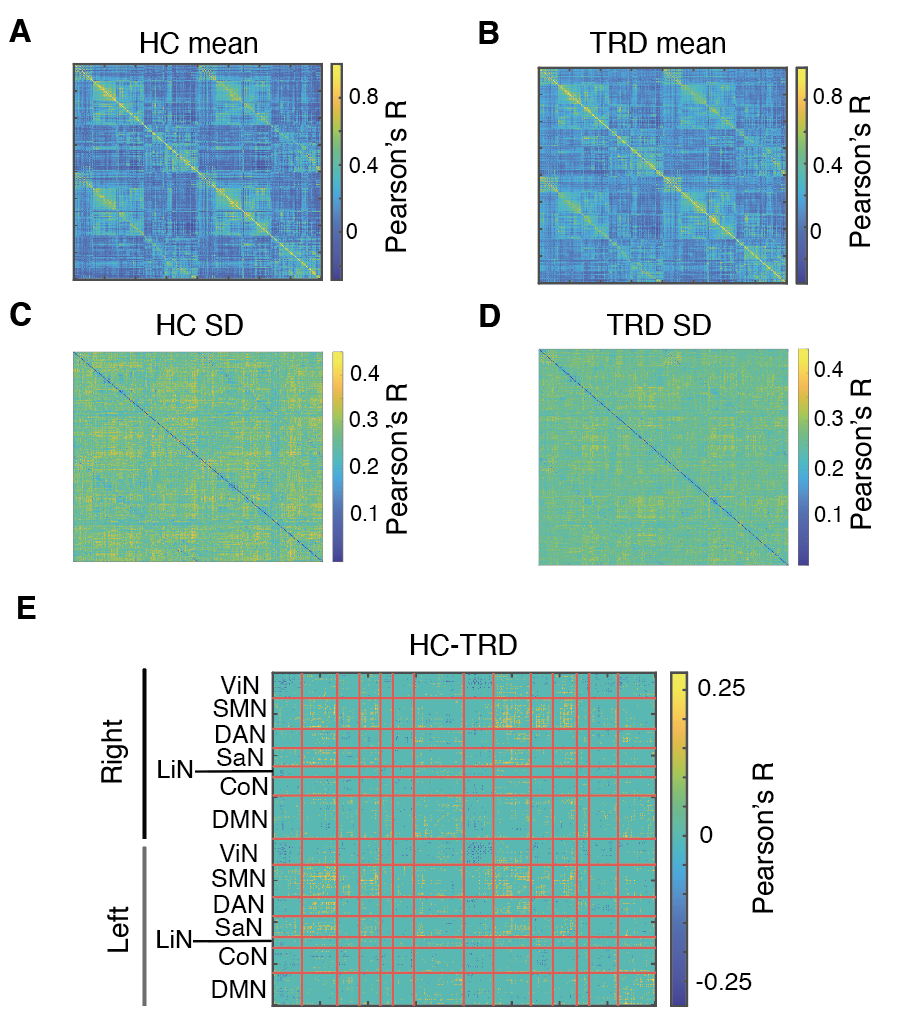


**Supplementary Figure S1.** Mean functional connectivity matrices derived from rs-fMRI data of **(A)** HCs and **(B)** patients with TRD. Standard deviation (SD) of functional connectivity matrices derived from rs-fMRI data of **(C)** HCs and **(D)** patients with TRD. **(E)** Subtraction matrix showing functional connectivity differences across both groups. Warm colors reflect connectivity decreases in patients, while cold colors reflect connectivity increases in patients (p<0.05 uncorrected, no group differences survived FDR multiple comparison correction). CoN = Control Network; DAN = Dorsal Attention Network; DMN = Default Mode Network; HC = healthy controls; LiN = Limbic Network; SaN = Salience Network; SMN = Sensorimotor network; TRD = patients with treatment resistant depression; ViN = Visual Network.


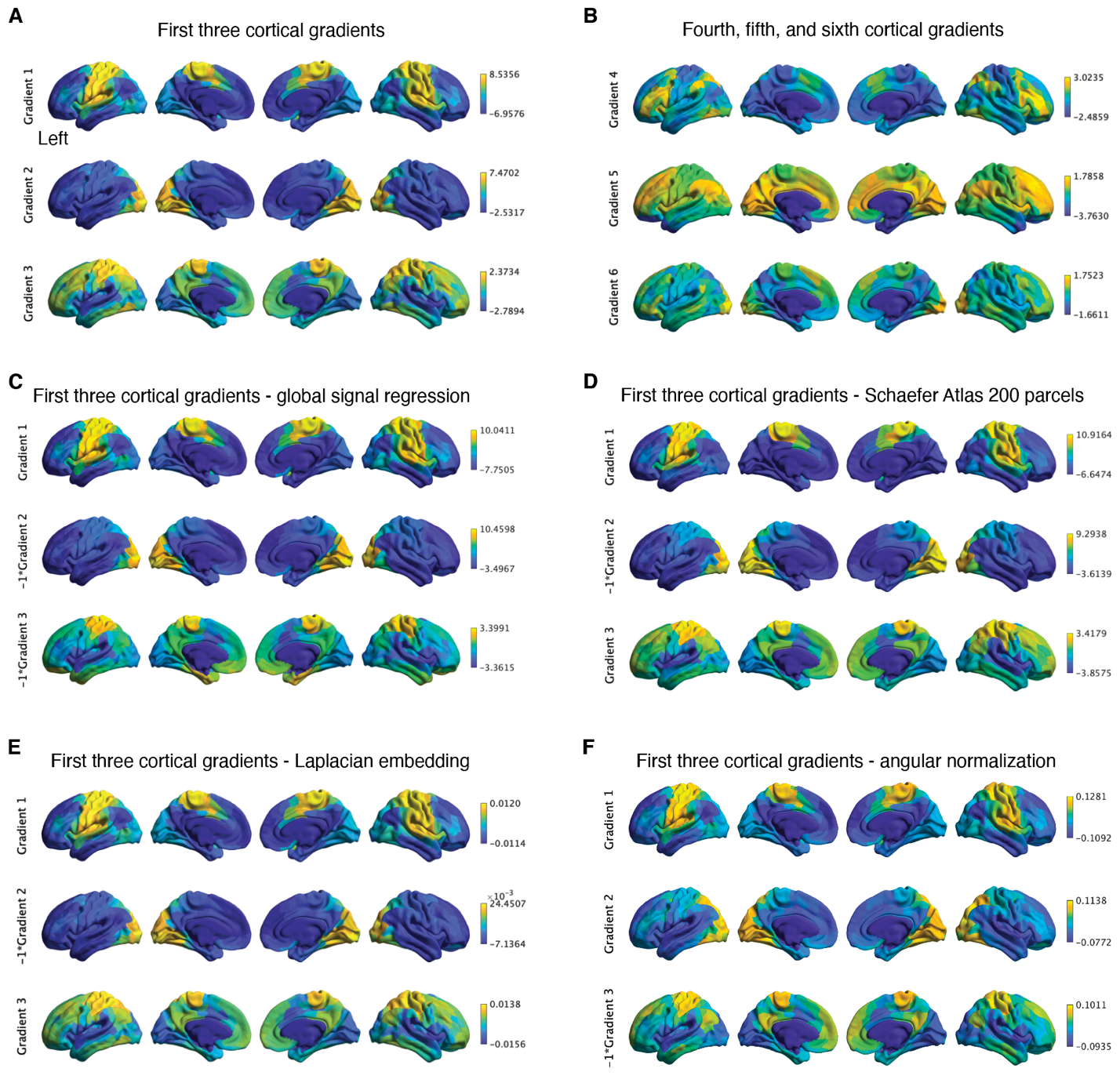


**Supplementary Figure S2. Cortical gradient extraction control analyses. (A)** Maps of cortical Gradients 1-3 as shown in the main findings. These gradients explained most of the variance in connectivity, followed by Gradients 5-6 **(B)**. First three cortical Gradients derived either through **(C)** global signal regressed rs-fMRI data; **(D)** Schaefer atlas with 200 parcels; **(E)** Laplacian embedding; or **(F)** angular normalization to generate the dissimilarity matrices. The sing assigned to gradients by the decomposition algorithm is arbitrary; we flipped the sign of some Gradients to better visualize the spatial similarity across corresponding gradient maps.
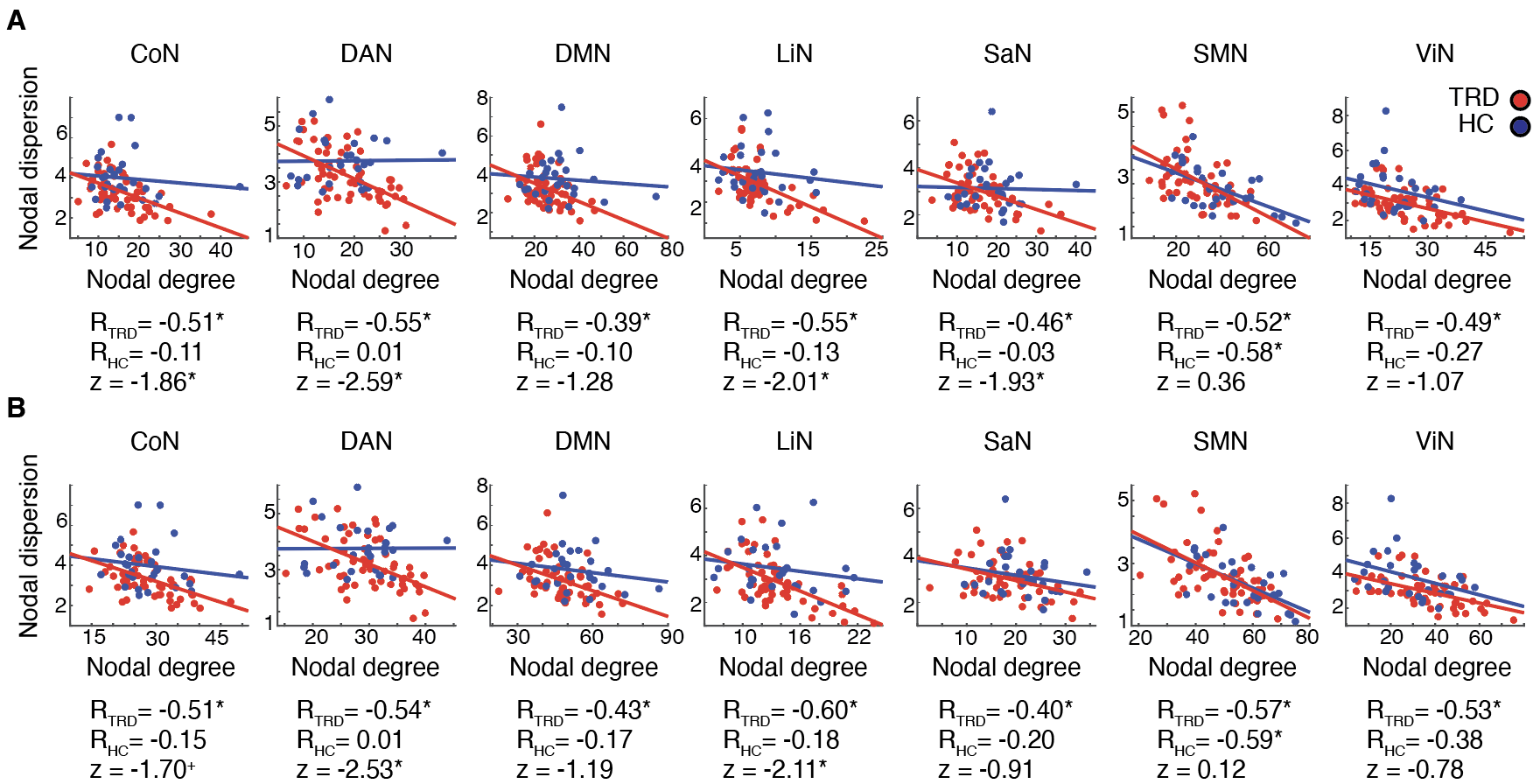


**Supplementary Figure S3. Within-network nodal dispersion and nodal degree control analyses.** Scatterplots reflecting the association between within-network nodal degree and within-network nodal dispersion separately for patients with TRD and HCs. Within-network nodal degree was repeatedly estimated by applying distinct thresholds when generating the weighted connectivity matrices. **(A)** Connectivity threshold set at 0.45. **(B)** Connectivity threshold set at 0.25. Pearson’s correlation coefficients are reported below the scatterplots for each group separately together with associated Fisher r-to-z tests for independent samples comparing the strength of the correlations across groups. CoN = Control Network; DAN = Dorsal Attention Network; DMN = Default Mode Network; HC = healthy controls; LiN = Limbic Network; SaN = Salience Network; SMN = Sensorimotor network; TRD = patients with treatment resistant depression; ViN = Visual Network. *p<0.05 FDR corrected, ^+^p<0.05 uncorrected


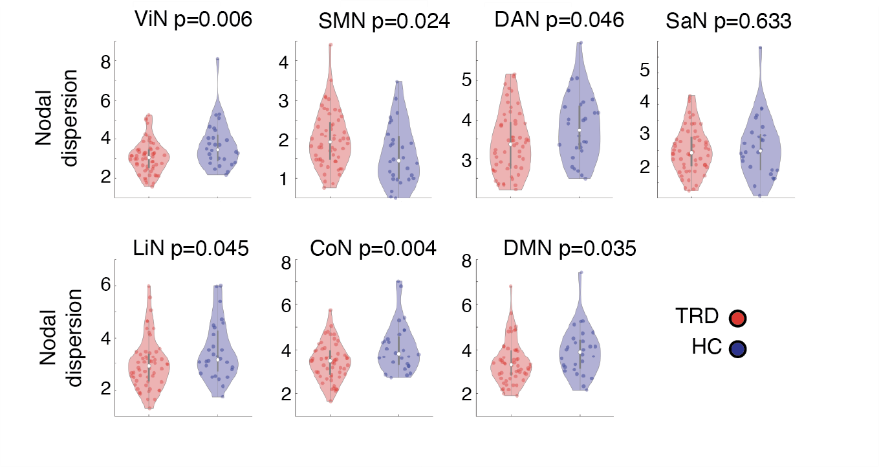


**Supplementary Figure S4. Within-network nodal dispersion derived using publicly available gradient maps.** Measures of nodal dispersion were derived by projecting individually decomposed functional connectivity maps into normative gradient maps derived from Margulies et al. 2016. Violinplots showing decreases in topographical within-network nodal dispersion in patients with TRD (red) when compared to HCs (blue). CoN = Control Network; DAN = Dorsal Attention Network; DMN = Default Mode Network; HC = healthy controls; LiN = Limbic Network; SaN = Salience Network; SMN = Sensorimotor network; TRD = treatment resistant depression; ViN = Visual Network.


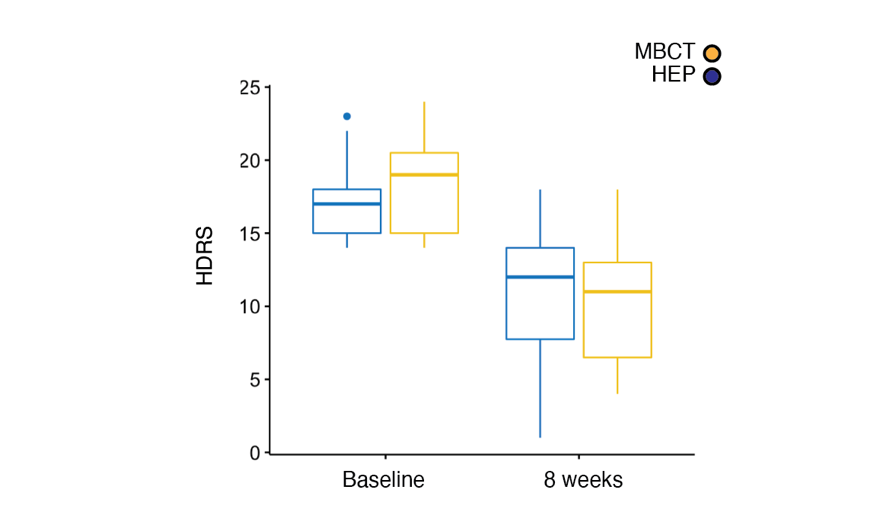


**Supplementary Figure S5. Changes in HDRS at 8 weeks post intervention with MBCT or HEP.** Patients with TRD show stronger reductions in HDRS scores at 8 weeks after completing a MBCT (orange) or a HEP intervention (blue). HDRS = Hamilton Depression Rating Score; HEP = health enhancement program; MBCT = Mindfulness-Based Cognitive Therapy.


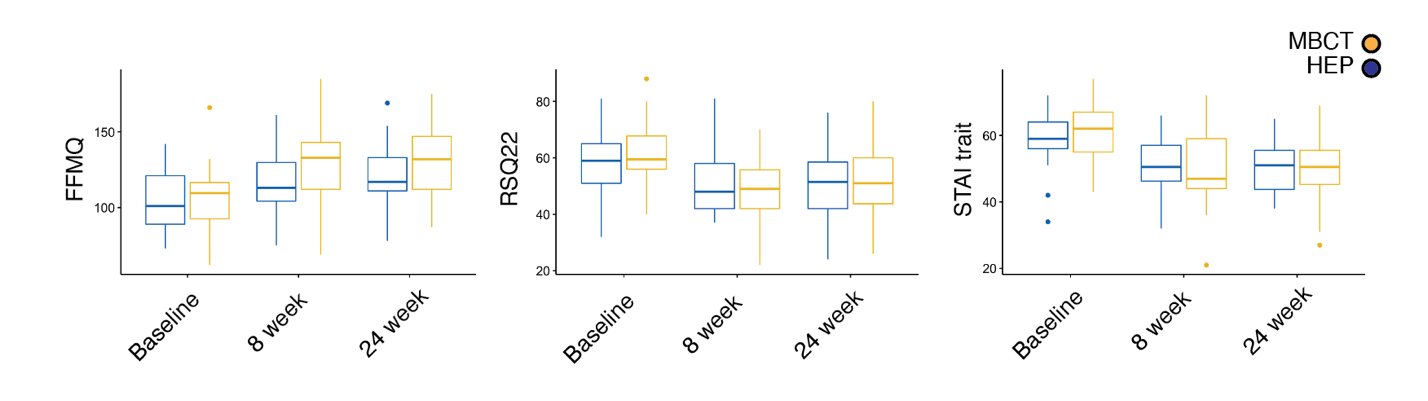


**Supplementary Figure S6. Changes in clinical scores with MBCT and HEP interventions.** Patients with TRD report higher levels of mindfulness, lower levels of depression, and lower levels of trait anxiety at 8 and 24 weeks after completing a MBCT (orange) or a HEP intervention (blue). HEP = health enhancement program; FFMQ = Five Facet; MBCT = Mindfulness-Based Cognitive Therapy; RSQ22 = Nolen-Hoeksema's Response Styles Questionnaire; STAI = State-Trait Anxiety Inventory.

|  | **Within-network nodal dispersion** | | | | | | |
| --- | --- | --- | --- | --- | --- | --- | --- |
|  | **CoN** | **DAN** | **DMN** | **LiN** | **SaN** | **SMN** | **ViN** |
| **Age** | β = -0.02  p = 0.21 | β = 0.00  p = 0.98 | β = -0.01  p = 0.71 | β = -0.00  p = 0.82 | β = 0.00  p = 0.94 | β = -0.01  p = 0.51 | β = 0.00  p = 0.83 |
| **Sex** | β = 0.17  p = 0.56 | β = 0.08  p = 0.80 | β = 0.19  p = 0.54 | β = -0.22  p = 0.49 | β = -0.18  p = 0.49 | β = -0.05  p = 0.86 | β = 0.20  p = 0.47 |
| **Number of spikes** | β = 0.00  p = 0.57 | β = -0.01  p = 0.29 | β = 0.00  p = 0.89 | β = -0.01  p = 0.14 | β = -0.00  p = 0.78 | β = 0.00  p = 0.51 | β = -0.01  p = 0.06 |

**Supplementary Table S1. Within-network nodal dispersion is not associated with head movement, age, or sex.** Seven separate multiple linear regression models were run in patients with TRD to assess the link between within-network nodal dispersion, used as the dependent variable, and age, sex, and numbers of movement spikes while scanned, defined as the number of volumes with a head framewise displacement higher than 0.5 mm. There were no significant associations. CoN = Control Network; DAN = Dorsal Attention Network; DMN = Default Mode Network; LiN = Limbic Network; SaN = Salience Network; SMN = Sensorimotor network; TRD = patients with treatment resistant depression; ViN = Visual Network.

| **i.** | **First six cortical gradients** | | | | | | |
| --- | --- | --- | --- | --- | --- | --- | --- |
|  | **ViN** | **SMN** | **DAN** | **SaN** | **LiN** | **CoN** | **DMN** |
| **TRD** | 3.7 (0.9) | 3.2 (0.9) | 3.9 (0.8) | 3.7 (0.8) | 3.6 (1.0) | 4.1 (0.8) | 4.2 (1.0) |
| **HC** | 4.5 (1.3) | 2.8 (0.9) | 4.3 (0.8) | 3.6 (0.9) | 4.0 (1.1) | 4.7 (1.1) | 4.5 (1.1) |
| **p** | 0.003* | 0.074 | 0.025 | 0.850 | 0.083 | 0.010* | 0.155 |
| **ii.** | **Global signal regression** | | | | | | |
|  | **ViN** | **SMN** | **DAN** | **SaN** | **LiN** | **CoN** | **DMN** |
| **TRD** | 3.5 (0.9) | 2.8 (0.8) | 3.6 (0.9) | 3.1 (0.7) | 3.3 (1.1) | 3.8 (1.0) | 3.7 (1.0) |
| **HC** | 4.3 (1.6) | 2.6 (0.8) | 4.3 (1.0) | 3.5 (0.9) | 4.2 (1.5) | 4.6 (1.7) | 4.3 (1.3) |
| **p** | 0.007* | 0.601 | 0.002* | 0.026* | 0.003* | 0.005* | 0.013* |
| **iii.** | **Schaefer Atlas 200 parcels** | | | | | | |
|  | **ViN** | **SMN** | **DAN** | **SaN** | **LiN** | **CoN** | **DMN** |
| **TRD** | 3.6 (1.3) | 3.2 (1.4) | 4.0 (1.5) | 3.8 (1.2) | 3.4 (1.5) | 4.3 (1.4) | 4.0 (1.4) |
| **HC** | 4.5 (1.7) | 2.9 (1.2) | 4.4 (1.3) | 3.5 (1.4) | 3.7 (1.5) | 4.7 (1.6) | 4.2 (1.2) |
| **p** | 0.007* | 0.299 | 0.227 | 0.401 | 0.353 | 0.283 | 0.416 |
| **iv.** | **Schaefer Atlas 1000 parcels** | | | | | | |
|  | **ViN** | **SMN** | **DAN** | **SaN** | **LiN** | **CoN** | **DMN** |
| **TRD** | 2.8 (0.9) | 2.1 (0.6) | 2.9 (0.7) | 2.5 (0.6) | 2.5 (0.8) | 3.0 (0.8) | 2.9 (0.8) |
| **HC** | 3.4 (1.1) | 2.0 (0.6) | 3.4 (0.8) | 2.7 (0.7) | 3.1 (1.0) | 3.6 (1.1) | 3.2 (0.8) |
| **p** | 0.002* | 0.562 | 0.003* | 0.218 | 0.004* | 0.013* | 0.043 |
| **v.** | **Laplacian embedding – values multiplied by 100** | | | | | | |
|  | **ViN** | **SMN** | **DAN** | **SaN** | **LiN** | **CoN** | **DMN** |
| **TRD** | 1.3 (0.2) | 1.3 (0.2) | 1.3 (0.2) | 1.3 (0.3) | 1.2 (0.2) | 1.3 (0.2) | 1.2 (0.2) |
| **HC** | 1.4 (0.2) | 1.3 (0.3) | 1.3 (0.2) | 1.3 (0.2) | 1.2 (0.2) | 1.3 (0.2) | 1.2 (0.2) |
| **p** | 0.080 | 0.150 | 0.266 | 0.379 | 0.514 | 0.968 | 0.234 |
| **vi.** | **Angular normalization – values multiplied by 100** | | | | | | |
|  | **ViN** | **SMN** | **DAN** | **SaN** | **LiN** | **CoN** | **DMN** |
| **TRD** | 6.8 (1.0) | 7.3 (1.2) | 7.9 (1.0) | 7.2 (1.0) | 5.9 (1.3) | 7.3 (0.9) | 7.1 (1.0) |
| **HC** | 6.9 (1.1) | 7.5 (1.3) | 8.4 (1.1) | 7.5 (1.1) | 6.0 (1.1) | 7.5 (1.2) | 7.5 (1.1) |
| **P** | 0.657 | 0.440 | 0.051 | 0.214 | 0.601 | 0.337 | 0.069 |

**Supplementary Table S2. Within-network nodal dispersion across methodological parameters.** Group-mean within-network nodal dispersion and standard deviation in brackets**.** We systematically assessed whether group differences in within-network nodal dispersion were affected by methodological parameters, such as: (i) including up to six cortical gradients when assessing the Euclidean distance between nodes; (ii) performing global signal regression on rs-fMRI data; (iii) using the Schaefer Atlas at a lower spatial resolution of 200 parcels; (iv) using the Schaefer Atlas at a higher spatial resolution of 1000 parcels; (v) applying Laplacian embedding to derive cortical gradients; or (vi) using angular normalization to generate the dissimilarity matrices. Raw p values are reported; *denotes p<0.05 FDR corrected for multiple comparisons. Significant FDR corrected reductions in within-network nodal degree in patients are highlighted in the orange cells. CoN = Control Network; DAN = Dorsal Attention Network; DMN = Default Mode Network; LiN = Limbic Network; SaN = Salience Network; SMN = Sensorimotor network; TRD = patients with treatment resistant depression; ViN = Visual Network.

| **Nodal dispersion** | **DMN** | **CoN** | **LiN** |
| --- | --- | --- | --- |
| **HDRS-17** | R = 0.12  p = 0.37 | R = 0.04  p = 0.74 | R = 0.13  p = 0.32 |
| **Nodal degree** | **DMN** | **CoN** | **LiN** |
| **HDRS-17** | R = 0.23  p = 0.08 | R = 0.37  p = 0.006 | R = -0.12  p = 0.35 |

**Supplementary Table S3. Association of clinical depression severity with nodal dispersion and nodal degree in TRD patients.** Nodal dispersion of the DMN, CoN, and LiN did not significantly correlate with clinical depression severity in TRD patients, as assessed through the HDRS-17. Within-network nodal degree of the CoN correlated positively with depression severity; DMN nodal degree displayed a trending positive correlation. CoN = Control Network; DMN = Default Mode Network; HDRS-17 = Hamilton Depression Rating Scale; LiN = Limbic Network; TRD = treatment resistant depression.

| **Change score** | **Group** | **Time** | **Group*Time** |
| --- | --- | --- | --- |
| **STAI trait** | F = 0.0  p = 0.96 | F = 19.2  p < 0.001 | F =0.5  p = 0.61 |
| **RSQ22** | F = 0.10  p = 0.78 | F = 10.9  p < 0.001 | F =0.6  p = 0.57 |
| **FFMQ** | F = 4.0  p < 0.05 | F = 11.6  p < 0.001 | F = 1.0  p = 0.36 |

**Supplementary Table S4. Repeated measurement ANOVA models assessing change in STAI trait, RSQ22, and FFMQ scores in patients.** Repeated measurement ANOVA models revealed a significant effect of time on scores of STAI trait, RSQ22, and FFMQ from baseline to 8 and 24 weeks. No significant group x time interactions were found. FFMQ = Five Facet Mindfulness Questionnaire; RSQ22 = Nolen-Hoeksema's Response Styles Questionnaire; STAI = State-Trait Anxiety Inventory.

**Supplementary References**

1. Eisendrath SJ, Gillung E, Delucchi KL, Zindel V, Nelson JC, Mcinnes LA, *et al.* (2016): A Randomized Controlled Trial of Mindfulness-Based Cognitive Therapy for Treatment-Resistant Depression Stuart. *Psychother Psychosom* 85: 99–110.

2. Ferri J, Eisendrath SJ, Fryer SL, Gillung E, Roach BJ, Mathalon DH (2017): Blunted amygdala activity is associated with depression severity in treatment-resistant depression. *Cogn Affect Behav Neurosci* 17: 1221–1231.

3. Segal Z v, Williams JMG, Teasdale JD (2013): Mindfulness-based cognitive therapy for depression, 2nd ed. *Mindfulness-Based Cognitive Therapy for Depression, 2nd Ed.* New York,  NY,  US: The Guilford Press.

4. MacCoon DG, Imel ZE, Rosenkranz MA, Sheftel JG, Weng HY, Sullivan JC, *et al.* (2012): The validation of an active control intervention for Mindfulness Based Stress Reduction (MBSR). *Behaviour Research and Therapy* 50.

5. Schaefer A, Kong R, Gordon EM, Laumann TO, Zuo X-N, Holmes AJ, *et al.* (2018): Local-Global Parcellation of the Human Cerebral Cortex from Intrinsic Functional Connectivity MRI. *Cerebral Cortex* 28: 3095–3114.

6. Yeo BTT, Krienen FM, Sepulcre J, Sabuncu MR, Lashkari D, Hollinshead M, *et al.* (2011): The organization of the human cerebral cortex estimated by intrinsic functional connectivity. *J Neurophysiol* 106: 1125–1165.

7. Bethlehem RAI, Paquola C, Seidlitz J, Ronan L, Bernhardt B, Consortium CCAN, Tsvetanov KA (2020): Dispersion of functional gradients across the adult lifespan. *Neuroimage* 222.

8. Margulies DS, Ghosh SS, Goulas A, Falkiewicz M, Huntenburg JM, Langs G, *et al.* (2016): Situating the default-mode network along a principal gradient of macroscale cortical organization. *Proceedings of the National Academy of Sciences* 113: 12574–12579.

9. Power JD, Mitra A, Laumann TO, Snyder AZ, Schlaggar BL, Petersen SE (2014): NeuroImage Methods to detect , characterize , and remove motion artifact in resting state fMRI. *Neuroimage* 84: 320–341.
